# Activation of autophagy by FOXO3 regulates redox homeostasis during osteogenic differentiation

**DOI:** 10.1080/15548627.2016.1203484

**Published:** 2016-08-17

**Authors:** M. C. Gómez-Puerto, L. P. Verhagen, A. K. Braat, E. W.-F. Lam, P. J. Coffer, M. J. Lorenowicz

**Affiliations:** a Center for Molecular Medicine, University Medical Center Utrecht, Utrecht, The Netherlands; b Regenerative Medicine Center, Uppsalalaan 8, Utrecht, The Netherlands; c Department of Surgery and Cancer, Imperial College London, Hammersmith Hospital Campus, London, UK

**Keywords:** autophagy, FOXO3, human mesenchymal stem cells (hMSCs), mitogen-activated protein kinase 8 (MAPK8/JNK), osteoblasts, reactive oxygen species (ROS)

## Abstract

Bone remodeling is a continuous physiological process that requires constant generation of new osteoblasts from mesenchymal stem cells (MSCs). Differentiation of MSCs to osteoblast requires a metabolic switch from glycolysis to increased mitochondrial respiration to ensure the sufficient energy supply to complete this process. As a consequence of this increased mitochondrial metabolism, the levels of endogenous reactive oxygen species (ROS) rise. In the current study we analyzed the role of forkhead box O3 (FOXO3) in the control of ROS levels in human MSCs (hMSCs) during osteogenic differentiation. Treatment of hMSCs with H_2_O_2_ induced FOXO3 phosphorylation at Ser294 and nuclear translocation. This ROS-mediated activation of FOXO3 was dependent on mitogen-activated protein kinase 8 (MAPK8/JNK) activity. Upon FOXO3 downregulation, osteoblastic differentiation was impaired and hMSCs lost their ability to control elevated ROS levels. Our results also demonstrate that in response to elevated ROS levels, FOXO3 induces autophagy in hMSCs. In line with this, impairment of autophagy by autophagy-related 7 (ATG7) knockdown resulted in a reduced capacity of hMSCs to regulate elevated ROS levels, together with a reduced osteoblast differentiation. Taken together our findings are consistent with a model where in hMSCs, FOXO3 is required to induce autophagy and thereby reduce elevated ROS levels resulting from the increased mitochondrial respiration during osteoblast differentiation. These new molecular insights provide an important contribution to our better understanding of bone physiology.

## Introduction

Adult bones undergo a continuous remodeling that starts with the resorption of mineralized bone matrix by osteoclasts followed by *de novo* bone formation by osteoblasts. Osteoblasts are terminally differentiated and short lived cells (approximately 3 mo), therefore bone growth and maintenance requires their constant replacement with new osteoblasts originating from pluripotent mesenchymal stem cells (MSCs).^1,2^ Commitment and differentiation of MSCs toward osteoblast starts with osteoprogenitor cells that generate pre-osteoblasts, which subsequently develop into mature osteoblasts.^3^ To ensure a sufficient energy supply necessary for differentiation, MSCs undergo a metabolic switch which involves lowering glycolysis and increasing mitochondrial respiration.^4^ The increased mitochondrial metabolism is usually accompanied by increase in the endogenous reactive oxygen species (ROS), a potentially deleterious by-product of mitochondrial respiration.^5,6^ To prevent accumulation of ROS the differentiating MSCs activate a very efficient antioxidant defense system, which is at least partially based on upregulation of antioxidant enzymes such as manganese-dependent superoxide dismutase (SOD2/MnSOD) and catalase.^4^ However, mechanistic details of this antioxidant control in MSCs are poorly understood.

Forkhead box O (FOXO) transcription factors play an important role in the cellular defense against oxidative stress. The FOXO family comprises 4 members: FOXO1, FOXO3, FOXO4 and FOXO6. They can modulate the antioxidant responses through the transcriptional activation of SOD2,^7^ catalase^8^ and glutathione peroxidase, and by regulation of cell cycle, DNA repair and lifespan.^9,10^ In response to oxidative stress FOXOs are phosphorylated by MAPK8, mitogen-activated protein kinase 14 (MAPK14/p38 α) and serine/threonine-protein kinase 4 (STK4/MST1), which results in translocation to the nucleus and transcriptional activation of target genes. ROS-activated MAPK8 phosphorylates FOXO4 on threonine 447/threonine 451 and on threonine 223/serine 226,^11,12^ while MAPK14 activated by doxorubicin-induced ROS phosphorylates FOXO3 on Ser7.^13^ On the other hand, activation of STK4 by increased levels of ROS results in phosphorylation of FOXO1 and FOXO3 at serine 112 and serine 207 respectively, disrupting their binding to 14-3-3 protein β/α (YWHAB/14-3-3), a conserved regulatory protein, and promoting FOXO translocation to the nucleus.^14^

FOXOs have also been implicated in the regulation of osteoblasts differentiation and the maintenance of skeletal homeostasis.^15^ Conditional deletion of *Foxo1, Foxo3* and *Foxo4* in mice resulted in increased oxidative stress in bone, osteoblast apoptosis and a decrease in the number of osteoblasts. Conversely, overexpression of a *Foxo3* transgene in mature osteoblasts decreased oxidative stress and osteoblast apoptosis and increased the rate of bone formation.^16^ Thus, FOXOs appear to play an important role in bone biology by modulating the oxidative defense of mature osteoblasts. However, the role of FOXOs antioxidant properties in generation of new osteoblasts remains unclear. In this study we investigated how FOXO3 maintains redox homeostasis in human MSCs during their differentiation to osteoblasts. Our data demonstrate that in hMSCs ROS induces phosphorylation of FOXO3 and its translocation to the nucleus. This novel ROS-dependent phosphorylation of FOXO3 at serine 294 is mediated by MAPK8 kinase. We also show that upon H_2_O_2_ treatment, activation of FOXO3 in hMSCs results in downregulation of ROS through the activation of autophagy. Finally, our results demonstrate the important role of autophagy in the control of oxidative stress during the osteoblastic differentiation of hMSCs.

## Results

### FOXO3 regulates ROS levels in hMSCs during osteoblast differentiation

**Figure 1.**
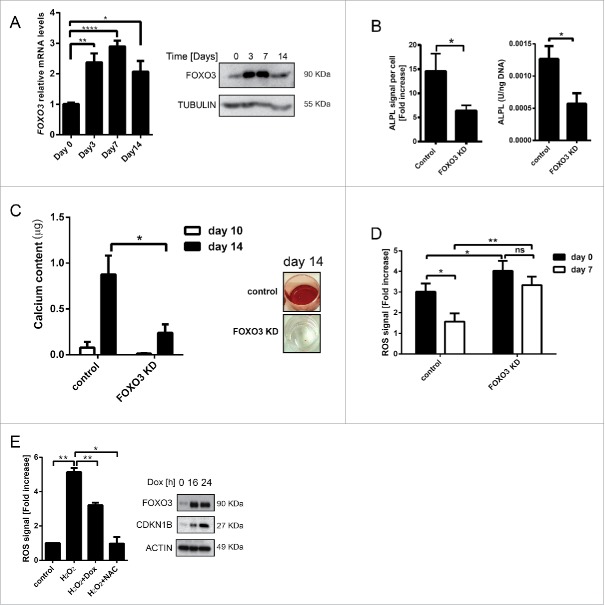
FOXO3 regulates ROS levels in hMSCs during osteoblast differentiation. (A) FOXO3 expression is upregulated during osteoblastic differentiation of hMSCs on mRNA and protein level. Primary BM-MSCs were differentiated to osteoblast and RNA or protein samples were collected at indicated time points. Left panel: *FOXO3* mRNA expression was analyzed by qRT-PCR. Data of 3 independent experiments performed in duplicates is presented as mean +/− SEM. * p < 0.05; **p < 0.001; ****p < 0.0001 . The data are presented as fold increases relative to day 0. Right panel: FOXO3 protein expression was analyzed by western blot. Representative results of at least 3 independent experiments are shown. (B) and (C) FOXO3 knockdown inhibits osteoblastic differentiation of hMSCs. Primary BM-MSCs were transfected with control or FOXO3 siRNA followed by a differentiation to osteoblasts. Subsequently, ALPL levels, ALPL activity or calcium content in the cultures were analyzed. (B) Left panel: The quantification of ALPL levels per cell at d 14 of osteoblastic differentiation is shown. Data of at least 5 independent experiments are presented as mean +/− SEM. * p < 0.05. The data are presented as fold increases relative to day 0. Right panel: The quantification of ALPL activity per ng DNA at d 10 of osteoblastic differentiation is shown. Data of at least 5 independent experiments performed in triplicates are presented as mean +/− SEM. * p < 0.05. (C) Left panel: The quantification of calcium content at d 10 and 14 of osteoblastic differentiation is shown. Data of 3 independent experiments performed in sextuplicates are presented as mean +/− SEM. * p < 0.05. Right panel: Representative pictures of Alizarin Red S stained monolayers at d 14 of osteoblastic differentiation are shown. (D) Knockdown of FOXO3 during osteoblastic differentiation of hMSCs decreases their ability to cope with increased ROS levels. Primary BM-MSCs were transfected with control or FOXO3 siRNA followed by a differentiation to osteoblasts. Cells were treated with H_2_O_2_ (50 μM) for 1 h at d 0 and 7 of osteoblastic differentiation and ROS levels were measured directly after the treatment as described in Materials and Methods. Data of 4 independent experiments are presented as mean +/− SEM. * p < 0.05; **p < 0.005. The data are presented as fold increases relative to d 0. (E) Activation of FOXO3 results in reduced ROS levels in hMSCs. Left panel: hMSCs- FOXO3-(A3) were treated with H_2_O_2_ (50 µM) for 1 h, with H_2_O_2_ (50 µM) combined with 16 h pretreatment with doxycycline (1 µg/ml) or with NAC (10 mM) for 2 h. ROS levels were measured directly after the treatment as described in Materials and Methods. Data of 3 independent experiments are presented as mean +/− SEM. * p < 0.05; **p < 0.005. The data are presented as fold increases relative to untreated control. Right panel: Western blot showing FOXO3 and CDKN1B levels after hMSCs-FOXO3-(A3) were treated with doxycycline (1 µg/ml) for 16 and 24 h. Representative results of at least 3 independent experiments are shown.

To investigate the role of FOXO3 in the regulation of ROS during osteoblast differentiation of hMSCs, the expression of FOXO3 was first analyzed. FOXO3 was upregulated on both the mRNA and protein level reaching maximum expression at d 7, with a decline at d 14 of differentiation (Fig. 1A). Importantly, FOXO3 knockdown resulted in the inhibition of osteoblastic differentiation of hMSCs, as measured by levels and activity of alkaline phosphatase (ALPL), an early marker of osteoblastic activity (Fig. 1B and Fig. S1A, B, C).^17^ To assess the effects of FOXO3 knockdown on matrix mineralization during osteoblastic differentiation, calcium deposition was measured using Alizarin Red S staining (Fig. 1C). The amount of calcium present in the cultures at d 10 and 14 of osteogenic differentiation was strongly reduced upon FOXO3 knockdown confirming the important role of FOXO3 in this process.

To explore whether FOXO3 knockdown-mediated inhibition of osteoblast differentiation was a result of the reduced ability of MSCs to manage increased oxidative stress, hMSCs were treated with H_2_O_2_ at d 0 and 7 of differentiation, and levels of ROS were measured. In line with an increase of FOXO3 expression at d 7 (Fig. 1A), hMSCs transfected with control siRNA showed a reduction in ROS levels at this time point (Fig. 1D). In contrast, FOXO3 knockdown resulted in elevated ROS levels at d 0 and d 7 of hMSCs differentiation to osteoblasts, indicating that FOXO3 is important for the regulation of ROS in these cells (Fig. 1D). To further corroborate these results, an immortalized hMSCs cell line (hMSCs-TERT) carrying doxycycline (DOX) inducible, constitutively active FOXO3 (further referred to as hMSCs-FOXO3-(A3)) was generated. The morphology and CD marker profile of these hMSCs is highly comparable to nontransduced bone marrow derived hMSCs (Fig. S2A and B). Furthermore, hMSCs-TERT have the ability to differentiate toward osteoblasts (Fig. S2C, E-G) and adipocytes (Fig. S2H), which confirms their hMSCs identity. Likewise, hMSCs-FOXO3-(A3) are also capable of osteogenic differentiation (Fig. S2D). Importantly, induction of FOXO3-(A3) expression by doxycycline treatment in hMSCs-FOXO3-(A3) resulted in increased levels of cyclin-dependent kinase inhibitor 1B (CDKN1B/p27^KIP1^), a FOXO-transcriptional target, indicating the activation of FOXO3-mediated transcription (Fig. 1E right panel; Fig. S1D). To establish whether FOXO3 can regulate ROS levels in hMSCs, hMSCs-FOXO3-(A3) were treated with H_2_O_2_ or with H_2_O_2_ preceded by pretreatment with DOX, and ROS levels were measured. As a control, hMSCs- FOXO3-(A3) were also treated with H_2_O_2_ combined with pretreatment with the ROS scavenger N-acetylcysteine (NAC), a synthetic precursor of intracellular cysteine and glutathione.^18,19^ Both the induction of FOXO3-(A3) signaling and treatment with NAC resulted in significant reduction of ROS levels in hMSCs (Fig. 1E, left panel). Taken together, these data indicate that FOXO3 is important in the regulation of augmented levels of ROS during hMSCs differentiation to osteoblasts.

### Activation of FOXO3 by oxidative stress is dependent on MAPK8 and MAPK11/12/14 MAP kinases

**Figure 2.**
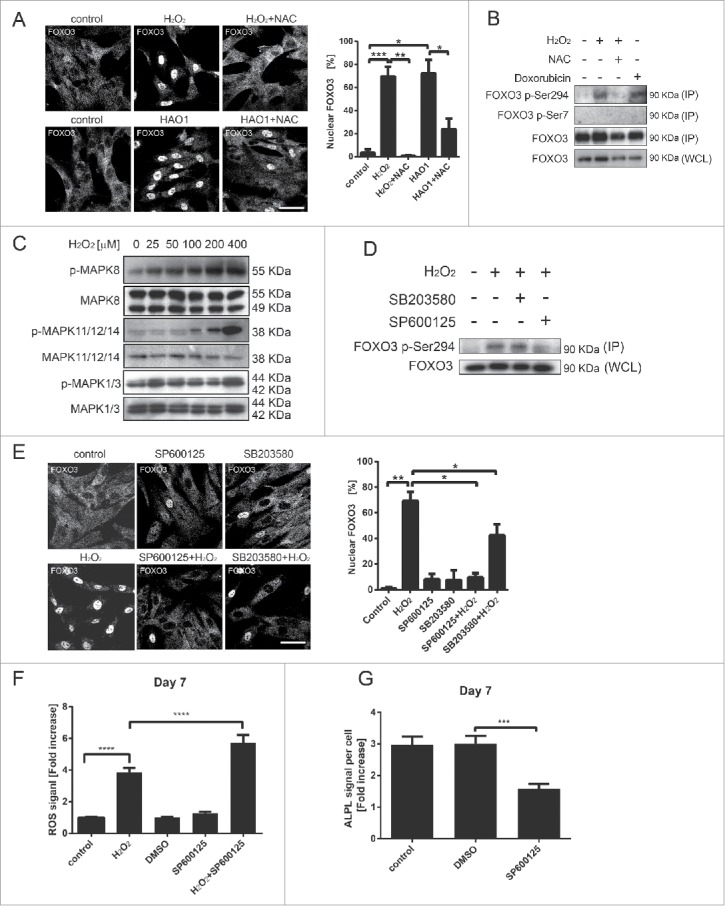
Activation of FOXO3 by oxidative stress is dependent on MAPK11/12/14 and MAPK8 MAP kinases. (A) FOXO3 translocates to the nucleus upon exposure to H_2_O_2_. hMSCs-TERT at 70% confluency were treated with H_2_O_2_ (400 µM) or with HAO1 (58 mU/ml) for 1 h and pretreated with NAC (10 mM) for 1 h where indicated. Left panel: Confocal microscopy images of FOXO3 localization are shown. Images are representative of 3 independent experiments. Scale bar is 50 µm. Right panel: Quantification of FOXO3 nuclear localization shown in (A). Data are presented as mean +/− SEM. * p < 0.05; **p< 0.01; *** p < 0.001. (B) Exposure to ROS induces phosphorylation of FOXO3 at the Ser294 residue. Primary BM-MSCs were treated with H_2_O_2_ (400 µM) or with doxorubicin (1 µM) for 1 h and pretreated with NAC (10 mM) for 1 h where indicated. FOXO3 was immunoprecipitated followed by western blot for FOXO3-Ser7 and FOXO3-Ser294. Representative results of 2 independent experiments are shown. (C) MAPK8 and MAPK11/12/14 are activated upon exposure of hMSCs to H_2_O_2_. hMSCs-TERT were treated for 1 h with indicated concentrations of H_2_O_2_ and directly lysed in sample buffer. Cell lysates were analyzed by western blot for presence of pMAPK8, pMAPK11/12/14 and ppMAPK1/3. Representative western blots of 3 independent experiments are shown. (D) The phosphorylation of FOXO3 is MAPK8 kinase dependent. Left panel: Primary BM-MSCs were treated with H_2_O_2_ (400 µM) for 1 h and/or with MAPK8 inhibitor SP600125 (25 µM) and MAPK11/12/14 inhibitor SB203580 (10 µM) for 2 h (including 1 h pre-treatment prior to addition of H_2_O_2_). FOXO3-Ser294 was immunoprecipitated followed by western blot for FOXO3. Representative results of 3 independent experiments are shown. (E) Inhibition of MAPK8 and MAPK11/12/14 prevents translocation of FOXO3 to the nucleus upon exposure to ROS. hMSCs-TERT were treated with H_2_O_2_ (400 µM) for 1 h and/or with MAPK8 inhibitor SP600125 (25 µM) and MAPK11/12/14 inhibitor SB203580 (10 µM) for 2 h (including 1-h pre-treatment prior to addition of H_2_O_2_). Left panel: Confocal microscopy images of FOXO3 localization are shown. Images are representative of 3 independent experiments. Scale bar is 50 µm. Right panel: Quantification of FOXO3 nuclear localization. Data are presented as mean +/− SEM. * p < 0.05; **p < 0.01. (F) Inhibition of MAPK8 kinase activity during hMSCs osteoblastic differentiation decreases their ability to control increased ROS levels. Primary BM-MSCs were differentiated to osteoblast in the presence or absence of SP600125 (25 µM). The differentiation medium containing the inhibitor was refreshed every day. At d 7 of osteoblastic differentiation cells were treated with H_2_O_2_ (25 µM) for 1 h. ROS levels were measured directly after the treatment as described in Materials and methods. Data of 3 independent experiments are presented as mean +/− SEM ****p < 0.0001. The data are presented as fold increases relative to untreated control. (G) Inhibition of MAPK8 kinase activity impairs differentiation of hMSCs to osteoblasts. Primary BM-MSCs were differentiated to osteoblast in the presence or absence of SP600125 (25 µM). The differentiation medium containing the inhibitor was refreshed every day. ALPL levels were analyzed by fluorescence microscopy (array scan). The quantification of ALPL levels per cell at d 7 of osteoblastic differentiation is shown. Data of 3 independent experiments are presented as mean +/− SEM. *** p < 0.0001. The data are presented as fold increases relative to d 0.

FOXO3 protein and mRNA expression is induced most prominently at d 7 and 10 of osteogenic differentiation when the highest ROS induction occurs.^20^ To determine whether ROS have a role in the regulation of FOXO3 activity in hMSCs, induction of high ROS levels during osteogenic differentiation was mimicked by exposing hMSCs to H_2_O_2_ for a short period of time. First, we examined the subcellular localization of FOXO3 by confocal microscopy after hMSCs were treated with H_2_O_2_ or hydroxyacid oxidase 1 (HAO1/GOX), an oxido-reductase that catalyzes the oxidation of glucose to hydrogen peroxide.^21,22^ Both H_2_O_2_ and HAO1 induced FOXO3 translocation to the nucleus, which could be abrogated by the pre-treatment of cells with NAC (Fig. 2A).

Phosphorylation of FOXO3 on Ser7 by the MAPK14 MAP kinase has been shown to promote its nuclear localization in response to doxorubicin,^13^ an anti-cancer drug that induces cytotoxicity by stimulating the production of intracellular free radicals. We therefore evaluated whether ROS-mediated nuclear translocation of FOXO3 is regulated by a similar MAP kinase-dependent mechanism in hMSCs. However, treatment of hMSCs with H_2_O_2_ or doxorubicin did not trigger FOXO3 Ser7 phosphorylation (Fig. 2B). In contrast, hMSCs doxorubicin and H_2_O_2_ induced the phosphorylation of FOXO3 at serine 294, previously identified as a target of MAPK11/12/14, MAPK8 and mitogen-activated protein kinase 1/3 (MAPK1/3, ERK1/2) MAP kinases.^13^ This novel ROS-dependent phosphorylation was decreased when cells were treated with NAC (Fig. 2B and Fig. S3A). To assess whether exposure to ROS has also an effect on other FOXO family members, hMSCs were treated with increasing doses of H_2_O_2_ and the phosphorylation of FOXO4 at threonine 223/serine 226, previously demonstrated to be a target of ROS-activated MAPK8 kinase,^12^ was evaluated. No changes in phosphorylation status of FOXO4 at this residue could be detected. Together our data show, that ROS promotes phosphorylation of FOXO3 at Ser294 and induces its translocation to the nucleus.

To identify the specific MAP kinase regulating FOXO3 Ser294 phosphorylation we analyzed the phosphorylation status of MAPK11/12/14, MAPK8 and MAPK1/3 MAP kinases after exposure of hMSCs to H_2_O_2_. H_2_O_2_ treatment induced phosphorylation of MAPK11/12/14 and MAPK8, but not MAPK1/3 (Fig. 2C; Fig. S3C), suggesting that increased ROS levels is sufficient to activate these 2 kinases in hMSCs. Next, the effect of MAPK11/12/14 or MAPK8 inhibition on ROS-mediated phosphorylation of FOXO3 on Ser294 was investigated. Phosphorylation of FOXO3 Ser294 was significantly decreased after treatment with MAPK8 kinase inhibitor (SP600125) and only moderately reduced by MAPK11/12/14 inhibition (SB203580) (Fig. 2D; Fig. S3D). In line with this, ROS-mediated translocation of FOXO3 to the nucleus was completely abrogated by treatment with MAPK8 kinase inhibitor and only partially inhibited by MAPK11/12/14 inhibitor treatment (Fig. 2E). Our results demonstrate that H_2_O_2_ can activate FOXO3 predominantly through MAPK8-induced Ser294 phosphorylation.

To determine whether inhibition of MAPK8 activity during osteoblastic differentiation affects the ability of hMSCs to control the ROS levels, hMSCs were treated with H_2_O_2_ and/or with SP600125 at d 7 of differentiation and ROS were measured. Despite the upregulated expression of FOXO3 at this time point of hMSCs differentiation (Fig 1A), MAPK8 inhibition resulted in increased ROS levels, suggesting that FOXO3 activity was compromised (Fig. 2F). Moreover, in the presence of SP600125 osteoblast differentiation was significantly less efficient (Fig. 2G; Fig. S3E-F), supporting a role for MAPK8 in this process.

### H_2_O_2_ treatment induces autophagy during hMSCs differentiation to osteoblasts

**Figure 3.**
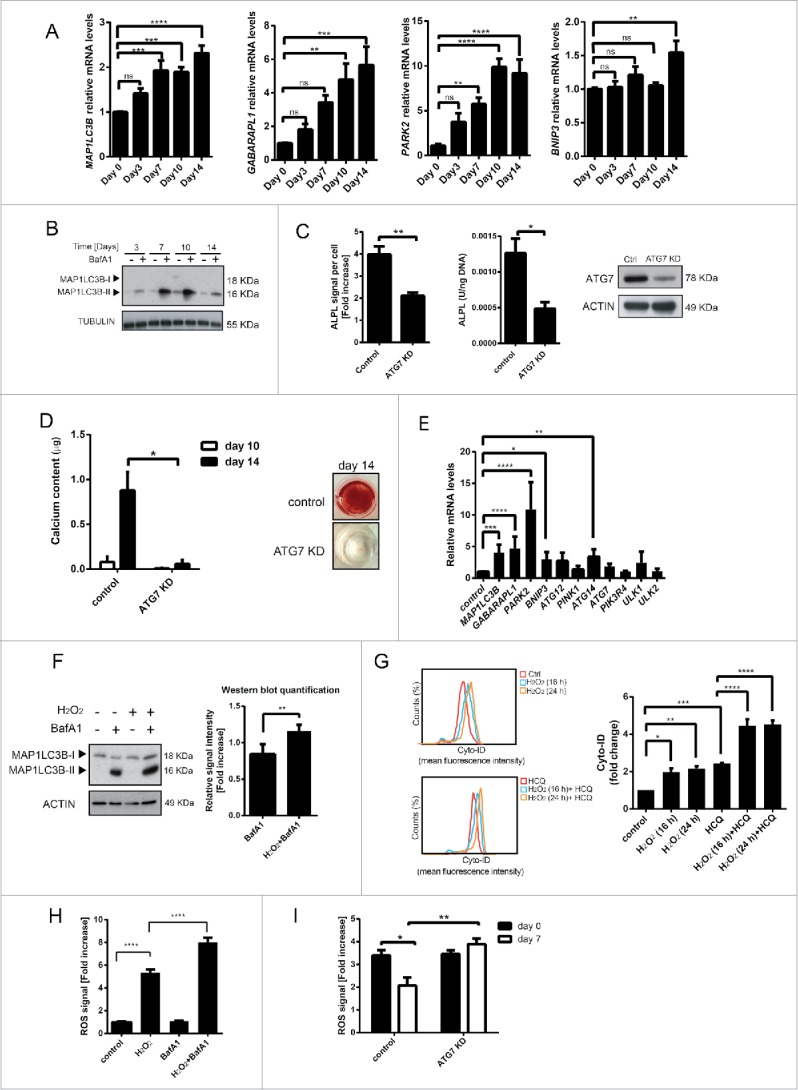
H_2_O_2_ treatment induces autophagy during hMSCs differentiation to osteoblasts. (A) Expression of autophagy genes is upregulated during osteogenic differentiation of primary BM-MSCs. Gene expression was analyzed by qRT-PCR. Quantification of data from 3 independent experiments perform in duplicates are shown as mean +/− SEM normalized for GAPDH. ** p < 0.005, ***p < 0.001 **** p < 0.0001. The data are presented as fold increases relative to day 0. (B) Western blot showing MAP1LC3BII levels during osteogenic differentiation of primary BM-MSCs. Actin is used as loading control. Representative results of 3 independent experiments are shown. (C) and (D) Inhibition of autophagy impairs differentiation of hMSCs to osteoblasts. (C) Left panel: hMSCs-TERT were transduced with control or ATG7 shRNA and differentiated to osteoblasts. ALPL levels were analyzed by fluorescence microscopy (array scan). The quantification of ALPL levels per cell at d 14 of osteoblastic differentiation is shown. Data of 3 independent experiments are presented as mean +/− SEM. ** p < 0.01. The data are presented as fold increases relative to d 0. Middle panel: Primary BM-MSCs were transfected with control or ATG7 siRNA followed by a differentiation to osteoblasts. Subsequently, ALPL activity was measured. The quantification of ALPL activity per ng DNA at d 10 of osteoblastic differentiation is shown. Data of at least 4 independent experiments performed in triplicates are presented as mean +/− SEM. * p < 0.05. Right panel: ATG7 levels analyzed by western blot. Actin is used as loading control. Representative results of 3 independent experiments. (D) Primary BM-MSCs were transfected with control or ATG7 siRNA followed by a differentiation to osteoblasts. Left panel: The quantification of calcium content at d 10 and 14 of osteoblastic differentiation is shown. Data of 3 independent experiments performed in sextuplicates are presented as mean +/− SEM. * p < 0.05. Right panel: Representative pictures of Alizarin Red S stained monolayers at d 14 of osteoblastic differentiation are shown. (E) H_2_O_2_ induces autophagy gene expression in hMSCs. hMSCs-TERT were treated with H_2_O_2_ (100 µM) for 24 h, lysed directly after the treatment and analyzed for the expression of genes involved in autophagy using qRT-PCR. Data of 3 independent experiments are presented as mean +/− SEM normalized for GAPDH. * p < 0.05; **p< 0.005, ***p < 0.001, **** p < 0.0001. The data are presented as fold increases relative to untreated control. (F) ROS induces upregulation of MAP1LC3B-II in hMSCs. hMSCs-TERT were treated with H_2_O_2_ (100 µM) with and without BafA1 (20 nM) for 16 h and lysed directly after the treatment . Left panel: MAP1LC3B-I and II levels analyzed by western blot. Actin is used as loading control. Representative results of 3 independent experiments. Right panel: Western-blot quantification of MAP1LC3B-II normalized for actin. Quantification of data from 3 independent experiments is shown as mean +/− SEM. **p < 0.005. The data are presented as fold increases relative to cells treated with BafA1. (G) Flow cytometry-based analysis of the quantification of autophagic vesicle content in hMSCs-TERT by means of the Cyto-ID dye after H_2_O_2_ (100 uM) treatment for 16 or 24 h with and without HCQ (20 uM) treatment for 16 h and analyzed directly after the treatment. Left panel: FACS plots, Right panel: quantification of the fold Cyto-ID mean fluorescence intensity upon different treatments. The data are presented as fold increases relative to untreated control. Data of 3 independent experiments are presented as mean +/− SEM. p < 0.05; **p< 0.005, ***p < 0.001, **** p < 0.0001. (H) Inhibition of autophagy results in increased ROS levels in hMSCs exposed to H_2_O_2_. hMSCs-TERT were treated for 4 h with BafA1 (20 nM) followed by 1 h treatment with H_2_O_2_ (50 uM). ROS levels were measured directly after the treatment as described in Materials and methods. Data of 3 independent experiments are presented as mean +/− SEM. **** p < 0.0001. (I) Knockdown of ATG7 during osteoblastic differentiation of MSC decreases their ability to control the increased ROS levels. Primary BM-MSCs were transfected with control or ATG7 siRNA followed by a differentiation to osteoblasts. Cells were treated with H_2_O_2_ (50 µM) for 1 h at d 0 and 7 of osteoblastic differentiation and ROS levels were measured directly after the treatment as described in Materials and methods. Data of 3 independent experiments are presented as mean +/− SEM. **p < 0.005, ***p < 0.001. The data are presented as fold increases relative to d 0.

Autophagy can play an antioxidant role by eliminating sources of excess ROS, which include damaged mitochondria and toxic aggregates.^23^ Importantly, we and others have previously demonstrated that FOXO transcription factors regulate this process.^24-26^ To establish whether autophagy may play a role in controlling ROS levels during osteogenic differentiation, the expression of components of the autophagy machinery known to be regulated by FOXO3 was determined.^24-26^ mRNA levels of microtubule-associated protein 1 light chain 3 β (*MAP1LC3B/LC3B*), GABA type A receptor associated protein like 1 (*GABARAPL1*), BCL2/adenovirus E1B 19kDa interacting protein 3 *(BNIP3)* and parkin RBR E3 ubiquitin protein ligase (*PARK2)* were all significantly upregulated in hMSCs undergoing osteogenic differentiation (Fig. 3A). However, increased expression of the autophagy machinery does not necessarily indicate an increase in autophagic flux. Therefore, autophagic turnover during osteogenic differentiation was studied by analyzing the levels of MAP1LC3B-II, a lipidated form of MAP1LC3B-I ubiquitin-like protein, which associates with autophagosomal membranes.^27^ An increase in MAP1LC3B-II is not a measure of autophagic flux on its own, since it can also indicate an inhibition of autophagosome clearance.^28^ Thus, to prevent lysosomal degradation and to block the fusion of autophagosomes with lysosomes, hMSCs were also treated with bafilomycin A1 (BafA1). MAP1LC3B-II was significantly upregulated during osteoblast differentiation, reaching the highest levels at d 7 and 10, which corresponds with the upregulation of FOXO3 during this process (Fig. 3B). Importantly, blocking autophagy by ATG7 knockdown, a critical component regulating the elongation and closure of the autophagosomal membrane,^28,29^ resulted in inhibition of osteoblast differentiation of hMSCs (Fig. 3C-D).

To investigate whether autophagy is triggered by elevated ROS levels in hMSCs, cells were treated with H_2_O_2_ for 24 h and the expression of genes critical for mitochondrial autophagy (mitophagy) and autophagic flux was determined. The expression of *MAP1LC3B, GABARAPL1, PARK2, BNIP3* and autophagy-related 14 (*ATG14*) were all significantly upregulated after exposure of hMSCs to H_2_O_2_ (Fig. 3E). Likewise, H_2_O_2_ induced accumulation of MAP1LC3B-II in the presence of BafA1 in hMSCs (Fig. 3F), indicating a ROS-dependent increase in autophagy flux. To further validate these findings, the autophagic flux of hMSCs was measured by flow cytometry (FACS) using Cyto-ID, a dye selectively labeling autophagic vacuoles.^30,31^ As expected, treatment of hMSCs with H_2_O_2_ increased the amount of autophagic vacuoles (Fig. 3G). Similarly, exposure to ROS combined with treatment with hydroxychloroquine (HCQ), an inhibitor of lysosomal function acting in a comparable way to BafA1 (Fig. S4)^27,32-35^ induced a higher autophagic flux in hMSCs (Fig. 3G). To demonstrate that autophagy is indeed necessary to downregulate ROS in hMSCs, cells were treated with BafA1 in combination with H_2_O_2_ and ROS levels were then determined. hMSCs treated with Baf1A and H_2_O_2_ showed a significant increase in ROS when compared to cells exposed to H_2_O_2_ alone (Fig. 3H). Likewise, blocking autophagy by ATG7 knockdown during osteoblast differentiation resulted in increased ROS levels at d 7 of differentiation in hMSCs exposed to H_2_O_2_ (Fig. 3I). Taken together, autophagy is an important anti-oxidant mechanism utilized by hMSCs to reduce ROS levels during osteogenic differentiation.

### FOXO3 reduces ROS levels by activation of autophagy in hMSCs

**Figure 4.**
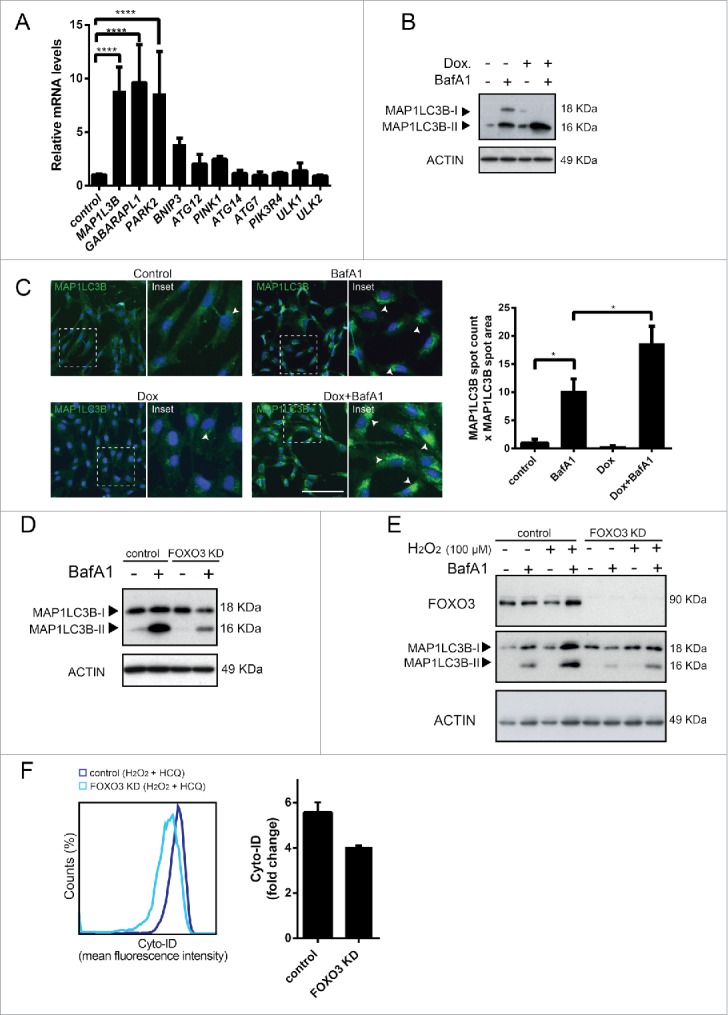
FOXO3 reduces ROS levels by activation of autophagy in hMSCs. (A) Activation of FOXO3 induces expression of genes involved in autophagy in hMSCs. hMSC-FOXO3-(A3) were treated with doxycycline (1 µg/ml) for 8 h and analyzed for the expression of indicated genes using qRT-PCR. Data of 3 independent experiments are presented as mean +/− SEM. ****p < 0.0001. The data are presented as fold increases relative to untreated control. (B) FOXO3 upregulates autophagy in hMSC. MAP1LC3B-I and II levels analyzed by western blot. Actin is used as loading control. (C) FOXO3 induces formation of MAP1LC3B positive autophagosomes. hMSC- FOXO3-(A3) were treated with doxycycline (1 µg/ml) for 16 h with and without BafA1 (20 nM). Cells were fixed and stained for MAP1LC3B. MAP1LC3B puncta were analyzed by fluorescence microscopy (array scan). Left panel: Representative pictures showing MAP1LC3B staining in green and DAPI positive nuclei in blue. Arrow heads indicate the MAP1LC3B positive autophagosomes. Right panel: Array scan quantification based on spot count and area of the spot. Quantification of data from 2 independent experiments performed in quadruplicates is shown as mean +/− SEM. *p < 0.05. (D) Knockdown of FOXO3 results in lower levels of MAP1LC3B-II in hMSC. hMSC-TERT were transfected with control or FOXO3 siRNA follow by BafA1 (20 nM) treatment for 16 h. MAP1LC3B-I and II levels analyzed by western blot are shown. Actin is used as loading control. Representative results of 3 independent experiments are shown. (E) Knockdown of FOXO3 inhibits H_2_O_2_-meadiated increase in MAP1LC3B-II levels. hMSC-TERT were transfected with control or FOXO3 siRNA followed by 16 h treatment with H_2_O_2_ (50 µM) in the presence or absence of BafA1 (20 nM) for 16 h and lysed directly after the treatment. FOXO3, MAP1LC3B-I and II levels analyzed by western blot are shown. Actin is used as loading control. Representative results of 3 independent experiments are shown. (F) Knockdown of FOXO3 results in a decrease of autophagocytic vesicles in hMSC. hMSC-TERT were transfected with control or FOXO3 siRNA followed by H_2_O_2_ (100 uM) treatment with and without HCQ (20 uM) for 16 h and analyzed directly after treatment. Autophagocytic vesicles were quantified using Cyto-ID dye-based flow cytometry analysis. Left panel: FACS plots, Right panel: quantification of the Cyto-ID mean fluorescence intensity. The data are presented as fold increase relative to untreated control. Data of 2 independent experiments are shown as mean +/− SEM.

To investigate whether ROS-induced autophagy is FOXO-dependent, the expression of genes regulating autophagy in hMSCs was evaluated upon conditional expression of constitutively active FOXO3. FOXO target genes including *MAP1LC3B, GABARAPL1* were significantly upregulated (Fig. 4A). mRNA levels of *PARK2* ^36-38^ a ubiquitin ligase indispensable for induction of mitophagy, the process important for clearance of ROS, were also significantly increased (Fig. 4A). To determine whether FOXO3 activation induces autophagic flux in hMSCs, hMSCs- FOXO3-(A3) were treated with DOX and BafA1 for 16h and MAP1LC3B-II was analyzed by western blot. Indeed, MAP1LC3B-II levels were significantly higher in hMSCs-FOXO3-(A3) treated with DOX and BafA1 when compared to cells treated with BafA1 alone (Fig. 4B; Fig. S5A). To study whether FOXO3 activation also promotes autophagosome formation, the number (spot count) and size (spot area) of MAP1LC3B-positive vesicles was analyzed by fluorescence microscopy in hMSCs-FOXO3-(A3) treated with DOX and BafA1 for 16 h^27,32,33^ (Fig. 4C). Conditional expression of FOXO3-(A3) increased the number and size of MAP1LC3B-positive punctate indicating increased formation of autophagosomes (Fig. 4C). Next, autophagic flux was analyzed after FOXO3 depletion. FOXO3 knockdown resulted in reduced BafA1-induced accumulation of MAP1LC3BII (Fig. 4D), further supporting the role of FOXO3 in the regulation of autophagy in hMSCs.

Finally, to validate that FOXO3 is necessary to trigger autophagy as a mechanism to reduce increased levels of ROS, we tested whether FOXO3 knockdown affects the autophagic flux in hMSCs treated with H_2_O_2_. Changes in autophagy were studied by analyzing MAP1LC3BII levels and by measuring autophagic vacuoles using Cyto-ID. Both MAP1LC3BII levels (Fig. 4E; Fig. S5B) and the number of autophagic vacuoles failed (Fig. 4F) to increase in the response to elevated ROS levels, when expression of FOXO3 was down regulated. Taken together, these data indicate that hMSCs induce FOXO3-mediated autophagy as a mechanism to reduce increased ROS levels that occur during osteoblastic differentiation.

## Discussion

Differentiation of hMSCs to osteoblasts is associated with an increase in mitochondrial content and activity, requiring a metabolic shift toward augmented oxidative phosphorylation.^20,39^ Increased mitochondrial respiration results in elevated levels of free oxygen radicals, which mediate oxidative damage to lipid, DNA and proteins and have been demonstrated to inhibit osteogenic differentiation of hMSCs.^16,40,41^ To prevent the damage induced by this mitochondrial-respiration by-product, hMSCs activate antioxidant defense mechanisms.^20^ Here for the first time, we provide molecular details underlying this antioxidant control. We demonstrate that in hMSCs elevated levels of ROS activate FOXO3 serine 294 phosphorylation in a MAPK8-dependent manner. Our data show that FOXO3 induces autophagy as a mechanism to downregulate elevated ROS in hMSCs, which is crucial for their proper differentiation to osteoblasts.

During osteoblast differentiation FOXO3 levels are elevated, corresponding to an increased capacity of hMSCs to deal with oxidative stress. Accordingly, ablation of FOXO3 in hMSCs results in augmented levels of ROS accompanied by a reduction in osteoblast differentiation. The inhibition of the osteoblast differentiation upon FOXO3 knockdown might be a consequence of hMSCs undergoing apoptosis, and indeed we observed elevated cleaved caspase-3 (CASP3) levels under this conditions (data not shown). These findings indicate that FOXO3 provides an important antioxidant defense mechanism for hMSCs and ensures generation of new osteoblasts that are crucial for maintaining proper balance in bone renewal. This is in line with previously demonstrated role of Foxos in the regulation of redox homeostasis in mature osteoblasts in mice.^16^ Our results show that FOXO3 knockdown partially affects the differentiation of hMCSs toward osteoblasts. We cannot exclude that other FOXO family members present in hMSCs contribute to this process. In line with this, FOXO1 knockdown has been reported to impair osteogenic differentiation of murine pre-osteoblastic cells MC3T3-E1.^42^

We found that ROS activate FOXO3 in hMSCs through induction of FOXO3 Ser294 phosphorylation and promoting nuclear translocation. ROS-mediated phosphorylation seems to be specific to FOXO3 in hMSCs, as no changes in phosphorylation of Thr223/Ser226 of FOXO4 were detected, the residues previously shown to be phosphorylated upon exposure of NIH3T3 cells to elevated ROS levels^12^. We cannot exclude the possibility of ROS-mediated FOXO1 phosphorylation, however no residues phosphorylated by ROS were identified for this FOXO family member so far. Thus, there are no tools available to evaluate whether phosphorylation of FOXO1 is modulated by ROS in hMSCs. ROS-mediated activation of FOXO3 is predominantly dependent on MAPK8 since both FOXO3 phosphorylation and translocation to the nucleus were abolished upon the MAPK8 inhibition. Previous reports have shown ROS-dependent phosphorylation of FOXO3 at Ser7 in MCF-7 cells mediated by MAPK11/12/14 kinase.^13^ Our data indicate that Ser7 is not phosphorylated upon the exposure of hMSCs to ROS. However, we demonstrate that ROS also induces the phosphorylation of MAPK11/12/14 MAP kinase albeit at higher concentration ranges (100-400 µM of H_2_O_2_). Inhibition of MAPK11/12/14 kinase also partially abrogates the translocation of FOXO3 to the nucleus upon exposure of hMSCs to the oxidative stress, suggesting that MAPK11/12/14 partially contributes to the ROS-mediated activation of FOXO3. It has been shown in vitro that MAPK11/12/14 can phosphorylate FOXO3 at Ser12, Ser294, Ser344 and Ser425.^13^ Thus, it is possible that higher levels of free radicals activate the MAPK11/12/14 kinase to enhance antioxidant activity of FOXO3, through phosphorylation of additional serine residues. FOXO3 Ser294 has been demonstrated to be a target of Ras-MAPK1/3 signaling, which is followed by downregulation of FOXO3 activity and induction of tumorigenesis.^43^ Here we show that ROS do not activate MAPK1/3 in hMSCs, indicating that it is the cellular and environmental context that defines the final outcome of FOXO3-mediated transcription.

FOXO transcription factors are known regulators of autophagy, which in turn contributes to the control of ROS homeostasis.^23,25,26^ Increased ROS levels induced expression of autophagy-related genes in hMSCs and importantly, a majority of these genes are also FOXO3 transcriptional targets. After FOXO3 depletion, ROS-induced autophagy was impaired, indicating that FOXO3-mediated regulation of autophagy is important for antioxidant defense in hMSCs. Likewise, impairment of autophagy by ATG7 knockdown resulted in a reduced capacity of hMSCs to regulate elevated ROS levels, together with a reduced osteoblast differentiation. This is in line with a recent finding in murine MSC, where inhibition of autophagy with chloroquine promoted H_2_O_2_ induced cell death.^44^ One of the possible mechanisms as to how autophagy could protect hMSCs against oxidative stress is by the clearance of damaged mitochondria.^23^ Indeed, Yue-Hua Yang et al. have demonstrated that treatment of mouse osteoblasts with H_2_O_2_ induced mitochondrial damage, as measured by decrease in the mitochondrial membrane potential. This resulted in engulfment of mitochondria into autophagosomal vacuoles and removal of these damaged organelles by osteoblasts.^45^

Autophagy has been also shown to be necessary for structural remodeling such as the developmental transitions observed during erythropoiesis.^46^ Thus, it is possible that FOXO3 regulates autophagy not only as a mechanism to downregulate ROS levels but also for structural remodeling of hMSCs during the osteogenic differentiation. It has been shown that the suppression of autophagy by FIP200 deletion led to decreased osteoblastic colony size and decreased osteoblast nodule size in bone marrow and primary calvaria cultures, respectively.^47^ These data suggested an inability of osteoblasts to switch from initial proliferation to mineralization upon FIP200 deletion.^47^

ATG7 knockdown resulted in only a partial inhibition of osteoblast differentiation. It is likely that hMSCs utilize multiple mechanisms to scavenge any excess of free radicals. It has been demonstrated that levels of antioxidant enzymes such as SOD2 and catalase are upregulated during osteogenic differentiation.^20^ FOXOs are also known regulators of ROS homeostasis through the transcriptional activation of SOD2^7,48,49^ and catalases.^8,50^ Indeed, our data demonstrate that both SOD2 and catalase mRNA are upregulated during osteogenic differentiation (Fig. S6A), however FOXO3 only induces expression of SOD2 and not catalase in hMSCs (Fig. S6B). It is possible that the transcriptional regulation of catalase in hMSCs is controlled by FOXO1 or FOXO4.^11^ Thus, FOXOs can govern the antioxidant defense of hMSCs during osteoblast differentiation through induction of antioxidant enzymes and increased autophagic flux. It would be interesting to further investigate whether autophagy and SOD2 are triggered simultaneously or if autophagy acts as an additional mechanism activated when SOD2 is not sufficient to reduce high ROS levels.

Taken together, our findings support a model where, in hMSCs, FOXO3 is required to induce autophagy and thereby reduce elevated ROS levels resulting from the increased mitochondrial respiration during osteoblast differentiation. These new molecular insights provide an important contribution to our better understanding of bone physiology. This may also serve as a basis to consider autophagy modulators as potential therapeutic treatment for diseases such as osteoporosis, where the redox homeostasis in bone is disrupted.

## Materials and methods

### Antibodies and reagents

The following anti-human antibodies were used: goat anti-actin from Santa Cruz Biotechnology (sc16160), mouse anti-MAP1LC3B was from Nanotools (5F10, 0231-100, recognizing the N-terminus of MAP1LC3B) and mouse anti-tubulin was from Sigma-Aldrich (T9026). Rabbit anti-Atg7 (8558), Rabbit anti-MAPK11/12/14 (9212), rabbit anti-phospho MAPK11/12/14 (9211s), rabbit anti-MAPK8 kinase (9252), rabbit anti-MAPK1/3 (9102) and rabbit anti-phospho MAPK1/3 (9101s) were from Cell Signaling Technology. Rabbit anti-phospho MAPK8 was from Millipore (559309-10T) while mouse anti-CDKN1B was from BD Biosciences (610241). A phospho-specific antibody against pSer294 of FOXO3 was generated by immunizing sheep with the peptide SKWPGpSPTSR, corresponding to residue 289-298 of FOXO3. The serum was affinity purified with the phospho-peptide and absorbed against the non-phospho peptide. It recognized the phosphorylated peptide at 250, 100 and 50 ng and not the non-phosphorylated peptide. Peroxidase-conjugated secondary antibodies were from Dako. Donkey anti-mouse cy5 secondary antibody was from Jackson (715-175-150). Hydroxychloroquine (HCQ), bafilomycin A1 (BafA1), H_2_O_2,_N-Acetylcystein (NAC), doxycycline (DOX), doxorubicin and glucose oxidase enzyme (HAO1) were obtained from Sigma-Aldrich. SB203580 (BML-EI286-0005) and SP600125 (BML-EI305-0050) were from Enzo Life Sciences.

### Cell culture and hMSCs differentiation

The human bone marrow MSCs (BM-MSCs) were isolated from bone marrow aspirate collected from a 3-y-old patient for routine diagnostic purposes, as approved by the institutional medical ethics committee. The BM aspirate was filtered through a 70 μm nylon cell strainer to remove cell aggregates and fragments of bone.

BM-MSCs were cultured in α-MEM (Gibco Invitrogen) supplemented with 10% HyClone serum (Gibco Invitrogen), 100 U/ml penicillin and 100 μg/ml streptomycin (Gibco Invitrogen), 2 mM L-glutamine (Gibco Invitrogen), 1 µg/ml Basic human Fibroblast Growth Factor (bFGF) (Gibco Invitrogen) and 200 µM L-ascorbic acid phosphate (Sigma-Aldrich). hMSCs-FOXO3-(A3) were maintained in the presence of 0.5 mg/ml G418 (Gibco Invitrogen).

To knock down the expression ATG7 or FOXO3, MSC were transfected with 10 nM of human ATG7 (L-020112-00-0005) or FOXO3 (L-003007000010) RNAi from Thermo Scientific using Lipofectamine RNAiMAX (Invitrogen). Cells were transfected 2 times with a 48 h interval between transfections. For osteogenic differentiation the culture medium was replaced with osteogenic differentiation medium 24 h after the last transfection. Where indicated, ATG7 was knockdown by lentiviral transduction using ATG7shRNA (Sigma-Aldrich).

For osteoblast differentiation, hMSCs were cultured during 14 d in DMEM (Gibco, Invitrogen) supplemented with 1% HyClone serum (Gibco Invitrogen), 200 µM L-glutamine (Invitrogen), 10 nM dihydroxyvitamin D3, 10 mM β-glycerolphosphate, 100 nM dexamethasone and 80 µg/ml ascorbic acid phosphate.^51^ The medium was replaced every 3-4 d.

### Alkaline phosphatase staining and activity assays

For ALPL staining cells were fixed in 4% formaldehyde at d 14 of differentiation. To verify the efficiency of differentiation the alkaline phosphatase was fluorescently labeled using the Vector Red Alkaline Phosphatase Substrate Kit (Vector Laboratories) according to manufacturer's instructions. To quantify the cell number, the cell nuclei were stained with DAPI. The fluorescence signal corresponding to ALPL levels and nuclear staining was measured using Cellomics ArrayScan VTI (Thermo Scientific; The Netherlands) with a 20x 0.45NA lens. The images were analyzed using Cellomics software. The average ALPL signal intensity per cell was quantified.^52^

For quantitative ALPL activity determination, cells were lysed in 0.5% (v/v) Triton X-100 in PBS for 30 min. ALPL activity was measured by conversion of the p-nitrophenyl phosphate Liquid Substrate System (Sigma-Aldrich). The absorbance was measured at 405 nm and corrected at 655 nm (Bio-rad, Hercules, CA, USA). Values were normalized to a standard ALPL measurement using serial dilutions of calf intestinal ALPI (Sigma-Aldrich) in 0.5% (v/v) Triton X-100 in PBS. The same cell lysate used to measure ALPL was stored at −80°C and subsequently used to determine the DNA content with the Quant-It PicoGreen kit (Invitrogen) according to the manufacturer's instructions.

### Calcium deposition

For qualitative assessment of matrix mineralization, the cell monolayer was fixed in 4% (w/v) paraformaldehyde, stained for 30 minutes with 0.2% (w/v) Alizarin red S solution (pH 4.2, Sigma-Aldrich) and the images of stained monolayers were taken. To quantify the calcium deposition, 10% cetylpyridinium was added for 60 minutes to extract the calcium-bound Alizarin. Absorbance was measured at 595 nm and corrected at 655 nm.

### Cell lines generation

Human bone marrow derived-MSC were immortalized through retroviral transduction with hTERT. Clonal MSC-hTERT lines were generated through limited dilution and were characterized for their morphology, proliferation, CD marker profile and in vitro differentiation potential.

To generate MSC line with inducible expression of constitutively active FOXO3 (FOXO3-A3) MSC-hTERT cells were transduced with lentiviral pInducer vector with neomycin resistance carrying FOXO3-A3. After selection the polyclonal cell population was tested for osteoblast differentiation potential.

### Immunoprecipitation and western blot analysis

For immunoprecipitation experiments hMSCs were plated on 100-mm plates and grown to 80 percent confluence. All subsequent steps were performed on ice. Cells were washed in phosphate-buffered saline (PBS; 137 mM NaCl, 2.7 mM KCl, 10 mM Na_2_PO_4_·2H_2_O and 2 mM KH_2_PO_4_ at pH 7.4) after the indicated treatments and incubated for 20 min with 750 μl of extraction buffer (20 mM Tris at pH 7.5, 150 mM NaCl, 10 mM EDTA, 1% Triton X-100, 0,1% SDS and 0,5% DOC) containing 10 mM NaF, 0.5 mM sodium orthovanadate, a Halt™ Protease Inhibitor Cocktail and a Halt™ phosphatase inhibitor cocktail (Thermo Scientific). The cell lysate was centrifuged for 10 minutes at 12,000 g and the supernatant was collected. The lysate was pre-incubated with protein A–Sepharose beads (GE Healthcare) for 1 h. After the removal of the beads, the lysate was incubated with anti-FOXO3 or FOXO3-Ser294 together with fresh protein A beads for 2 h. Subsequently the beads were washed 3 times in the same extraction buffer. Dried protein A beads were boiled for 5 min with 30 μl Laemmli sample buffer. The supernatant was then collected and subjected to western blotting.

Western blot analysis was performed using standard techniques. In brief, hMSCs were lysed in Laemmli buffer (0.12 M Tris HCL pH 6.8, 4% SDS, 20% glycerol, 35 mM β–mercaptoethanol) and boiled for 5 min. Equal amounts of total lysate were analyzed by SDS-polyacrylamide gel electrophoresis. Proteins were transferred to polyvinylidene difluoride (PVDF) membrane (Millipore) and incubated with the appropriate antibodies according to the manufacturer's instructions. Membranes were washed, incubated with appropriate peroxidase-conjugated secondary antibodies and developed by ELC (Amersham Pharmacia).

### Immunofluorescence analysis

MSC-TERT were plated on glass coverslips, fixed directly after the treatment in 0.1 M phosphate buffer containing 4% paraformaldehyde for 15 min at room temperature and permeabilized with 0.1% Triton X-100 for 5 min. Thereafter, cells were incubated with 2% BSA for 30 min followed by 1 h incubation with a rabbit anti-FOXO3 antibody (Cell Signaling) and subsequent incubation with a goat-anti-rabbit-Ig antibody labeled with Alexa 488 (Molecular Probes). For nuclear staining DAPI was used. Images were recorded on a Zeiss LSM 700 confocal microscope (Germany). The quantification of FOXO3 positive nuclei was done using the ImageJ Plugin the “Cell counter.” The FOXO3 positive nuclei was calculated as percentage of total nuclei labeled with DAPI.

### Array scan imaging

Doxycycline inducible hMSCs- FOXO3-(A3) were plated in a 96-well plate and treated with BafA1 (20 nM) in the presence or absence of DOX (1 ug/ml). Cells were then washed twice with PBS and fixed with cold methanol for 10 min at −20°C. Subsequently, cells were washed twice with PBS and incubated for 1 h with MAP1LC3B antibody at a dilution of 1:50 in PBS with 1% bovine serum albumin (BSA). Cells were then washed with PBS with 1% BSA and incubated for 1 h with donkey anti-mouse Cy5 secondary antibody at a dilution of 1:200 in PBS with 1% BSA. Next, cells were washed twice with PBS and stained with DAPI (1 μg/ml) for 5 min at room temperature. Fifteen images per well were captured in both DAPI and Cy5 channels on a Cellomics ArrayScan VTI (Thermo Scientific; The Netherlands) using a 20 × 0.45NA lens. Images were analyzed using the Cellomics SpotDetector V4 Bioapplication. Autofocussing was carried out on DAPI-stained nuclei. Exposure times were fixed for each individual experiment. Each nucleus was identified as a primary object in the DAPI channel, MAP1LC3B spots were detected within a 24 pixel mask defined around the nucleus. SpotCountPerObject and SpotTotalAreaPerObject were reported and used to determine changes in MAP1LC3B spot morphology.

### Flow cytometry analysis

hMSCs were treated with HCQ (20 µM) and H_2_O_2_ (100 µM) or DOX (1µg/ml). Cells were then trypsinized and incubated in α-MEM with Cyto-ID Autophagy Detection dye (Enzo Life Sciences, ENZ-51031-0050) at a dilution of 1:500 for 25 min at 37°C. Subsequently, cells were washed and analyzed by flow cytometry. All data were analyzed using FlowJo software.

### Reactive oxygen species measurements

hMSCs were incubated for 45 min at 37°C with 20 μM of H2DCFDA (Life Technologies) in PBS with 10% of HyClone serum. After undergoing different treatments, cells were treated with PrestoBlue (Life Technologies) according to manufacturer's protocol to determine cell viability. ROS signal per cell was measured. Measurements were performed using Specramax spectrophotometer (Molecular Devices, United Kingdom).

### Quantitative real-time PCR

RNA was isolated using RNeasy kit (Qiagen) according to the manufacturer's protocol. cDNA was generated by reverse transcribing 1 μg of total RNA with SuperScript III reverse transcriptase (Invitrogen Life Technologies). Quantitative real time PCR was subsequently performed using a Biorad Icycler (Bio-Rad, The Netherlands) with primer pairs for the indicated gene. Quantification was performed relative to the levels of the housekeeping gene glyceraldehyde-3-phosphate dehydrogenase (GAPDH) and normalized to time point 0 or control conditions. The data analysis was performed using the 2−ΔΔCT method.^53^ The primer sequences are listed in the Supplementary Table 1.

### Statistical analysis

Unpaired 2-sided Student *t* test or one way ANOVA was used to calculate statistical differences. A P-value of <0.05 was considered statistically significant.

## Supplementary Material

Supplementary files

## Abbreviations

ALPL: alkaline phosphatase, tissue-nonspecific isozyme
ATG7: autophagy-related 7
ATG14: autophagy-related 14
BafA1: bafilomycin
BNIP3: BCL2/adenovirus E1B 19kDa interacting protein 3
CASP3: caspase-3
CDKN1B: cyclin-dependent kinase inhibitor 1B
DOX: doxycycline
FOXO: forkhead box O
GABARAPL1: GABA type A receptor associated protein like 1
GAPDH: glyceraldehyde-3-phosphate dehydrogenase
HAO1: hydroxyacid oxidase 1
HCQ: hydroxychloroquine
hMSCs: human mesenchymal stem cells
MAPK 1/3: mitogen-activated protein kinase 1/3
MAPK8: mitogen-activated protein kinase 8
MAPK 11/12/14: mitogen-activated protein kinase 11/12/14
MAP1LC3B/LC3B: microtubule-associated protein 1 light chain 3 β
NAC: N-acetylcystein
PARK2: parkin RBR E3 ubiquitin protein ligase
ROS: reactive oxygen species
SOD2: superoxide dismutase 2
STK4: serine/threonine-protein kinase 4
