# Supplementary material for: Activation of autophagy by FOXO3 regulates redox homeostasis during osteogenic differentiation
Source: Autophagy. 2016 Aug 17;12(10):1804–16. doi: 10.1080/15548627.2016.1203484 (PMC5079670; doi:10.1080/15548627.2016.1203484)
Supplement: Supplementary files [file kaup-12-10-1203484-s001.zip › 2015AUTO0617R3-s02.pdf]

Supplementary Figure Legends

Figure S1. FOXO3 knockdown efficiency. (**A**) Western-blot comparing FOXO3 levels during osteoblast differentiation after primary BM-HsMSC were transfected with control siRNA or siRNA targeting *FOXO3*. Tubulin is used as loading control. Representative results of 3 independent experiments are shown. (**B and C**) Verification of FOXO3 knock down specificity. (**B**) Western blot and (**C**) qRT-PCR analysis of FOXO3, FOXO1 and FOXO4 expression in primary BM-HsMSC transfected with control siRNA or siRNA targeting FOXO3. Representative results of 3 independent experiments are shown. For western blot actin and HSP90AA1/HSP90 (heat shock protein 90kDa alpha family class A member 1) were used as a loading control. (**D**) Western-blot showing FOXO3 and CDKN1B levels after BM-HsMSCs-TERT and HsMSCs-FOXO3-(A3) were treated with doxycycline (1 µg/ml) at the indicated times.

Figure S2. Characterization of the HsMSC-TERT line. (**A**) Phase contrast microscopy pictures comparing the morphology of BM-HsMSCs-TERT and nontransduced primary bone marrow derived human MSCs (BM-HsMSCs). Scale bar: 20 µm. (**B**) Flow cytometry-based characterization of cord blood, BM-HsMSCs and BM-HsMSCs-TERT for the indicated hematopoietic cell, endothelial cell and MSC markers. (**C-G**) BM-HsMSCs-TERT, primary BM-HsMSCs and HsMSCs-FOXO3-(A3) have similar differentiation potential towards osteoblasts. (**C**) The quantification of ALPL levels per cell at day 14 of osteoblastic differentiation is shown. Data of at least 3 independent experiments are presented as mean +/- SEM. *, $p < 0.05$. The data are shown as fold increases relative to day 0. Left panel: ALPL signal for BM-HsMSCs-TERT. Right panel: ALPL signal for BM-

HsMSCs. (**D**) The quantification of ALPL levels per cell at day 14 of HsMSCs-FOXO3-(A3) osteoblastic differentiation is shown. Data of at least 3 independent experiments are presented as mean +/- SEM. *, p< 0.05. The data are shown as fold increases relative to day 0. (**E**) The quantification of ALPL activity per ng DNA at day 10 of osteoblastic differentiation is shown. Data of 3 independent experiments performed in sextuplicates are presented as mean +/- SEM. (**F**) Left panel: Quantification of Alizarin Red S staining absorbance at day 16 of osteoblastic differentiation is shown. Data of 3 independent experiments performed in 12 technical replicates are presented as mean +/- SEM. Middle panel: The quantification of calcium content at day 16 of osteoblastic differentiation is shown. Data of 3 independent experiments performed in 12 technical replicates are presented as mean +/- SEM. Right panel: Representative pictures of Alizarin Red S-stained monolayers at day 16 of osteoblastic differentiation are shown.

Figure S3. Characterization of the HsMSC-TERT line. (**A**) *COL1A1*/collagen mRNA expression during osteogenic differentiation of BM-HsMSCs (left panel) and BM-MSCs-HsTERT (right panel) was analyzed by qRT-PCR. The data are shown as fold increases relative to day 0. Data of 3 independent experiments performed in duplicates is presented as mean +/- SEM. (**B**) BM-MSCs-HsTERT differentiate to adipocytes. Nile red levels per cells are shown. Data of at least 3 independent experiments are presented as mean +/- SEM. *, p< 0.05.

Figure S4. Activation of FOXO3 by oxidative stress is dependent on MAPK11/12/14 and MAPK8. (**A**) Quantification of western blot from figure 2B. FOXO3 p-Ser294 was normalized for total FOXO3. The data are shown as fold increases relative to untreated

control and presented as mean +/- SEM. (**B**) **The** FOXO4 p-T223/S226 phosphorylation site is not affected upon exposure to increasing concentrations of H_2O_2. MSCs-HsTERT were treated for 1 h with the indicated concentrations of H_2O_2 and lysed directly after treatment. Cell lysates were analyzed by western blot for the presence of FOXO4 p-T223/S226. Upper panel: Representative western blots of 2 independent experiments are shown. Lower panel: Western blot quantification of FOXO4 p-T223/S226 normalized for tubulin and total FOXO4 is shown as fold increases relative to untreated control and presented as mean +/- SEM. Tubulin was used as a loading control. (**C**) Quantification of western blot from figure 2C. p-MAPK8, p-MAPK11/12/14 and p-MAPK1/3 were normalized for MAPK8, MAPK11/12/14 and MAPK8, respectively. The data are shown as fold increases relative to untreated control and presented as mean +/- SEM. Tubulin was used as a loading control. (**D**) Quantification of western blot from figure 2D. FOXO3 p-Ser294 was normalized for total FOXO3. The data are shown as fold increases relative to untreated control and presented as mean +/- SEM. (**E and F**) Inhibition of MAPK8 kinase activity impairs differentiation of hMSCs into osteoblasts. Primary BM-HsMSCs were differentiated into osteoblast in the presence or absence of SP600125 (25 µM). The differentiation medium containing the inhibitor was refreshed every day. (**E**) The quantification of ALPL activity per ng DNA at day 0 and 10 of osteoblastic differentiation is shown. Data of 2 independent experiments performed in sextuplicates are presented as mean +/- SEM. (**F**) Left panel: The quantification of calcium content at day 10 and 14 of osteoblastic differentiation is shown. Data of 2 independent experiments performed in sextuplicates are presented as mean +/- SEM. Right panel: Representative pictures of Alizarin Red S-stained monolayers at day 14 of osteoblastic differentiation are shown.

Figure S5. BafA1 and HCQ block turnover of MAP1LC3B-II in BM-HsMSCs and BM-HsMSCs-TERT. Western blot showing MAP1LC3B-I and MAP1LC3B-II levels in BM-HsMSCs and BM-HsMSCs-TERT after overnight treatment with BafA1 (20 nM) and HCQ (20 µM). Actin is used as a loading control.

Figure S6. Western blot quantifications. (**A**) Quantification of western blot from figure 4B. MAP1LC3B-II was normalized for actin. Quantification of data from 3 independent experiments is shown as mean +/- SEM. The data are presented as fold increases relative to cells treated with BafA1. (**B**) Quantification of western blot from figure 4E. MAP1LC3B-II was normalized for actin. Quantification of data from 3 independent experiments is shown as mean +/- SEM. The data are presented as fold increases relative to cells treated with BafA1.

Figure S7. FOXO3 induces *SOD2* expression in HsMSCs. (**A**) *SOD2* expression is upregulated during osteogenic differentiation of primary BM-MSCs. mRNA levels of *SOD2* (left panel) and *CAT* (catalase) (right panel) during osteogenic differentiation as measured by qRT-PCR are shown. The data are shown as fold increases relative to day 0. Quantification of data from 3 independent experiments performed in duplicates is shown as mean +/- SEM. *, $p<0.05$; **, $p<0.01$; ****, $p<0.0001$. (**B**) FOXO3 induces expression of SOD2 in HsMSC. HsMSCs-FOXO3-(A3) were treated with doxycycline (1 µg/ml) for 8 h and analyzed for the expression of the indicated genes using qRT-PCR. Data of 3 independent experiments are presented as mean +/- SEM. **, $p< 0.005$.

Supplementary Material and Methods

Flow cytometry analysis

For CD marker profiling cells were trypsinized, washed with PBS containing 2% fetal bovine serum (FACS buffer) and stained with anti-PECAM1/CD31, anti-PTPRC/CD45, anti-NT5E/CD73, anti-THY1/CD90 (BD Biosciences, 566177, 560566, 561258, 555595), and anti-ENG/CD105 (Southern Biotech, 9811-01) antibodies. After staining cells were resuspended in FACS buffer containing 1 µg/ml propidium iodide for dead cell exclusion and measured on a FACS Canto II (BD Biosciences, Breda). Data analysis was performed using FlowJo version 7.6 FlowJo LLC).

Adipocytic differentiation of HsMSCs

To induce differentiation of HsMSCs to adipocytes, cells were cultured in adipogenic induction medium, containing α-MEM (Gibco Invitrogen, 22571038) supplemented with 10% fetal bovine serum, 0.1 µM dexamethasone (Sigma-Aldrich, D4902), 0.5 mM isobutyl methyl xanthine (Sigma-Aldrich, I5879), 10 µg/ml insulin (Sigma-Aldrich, I3769), 100 µM indomethacin (Sigma-Aldrich, I7378) for 2 days.[47] Subsequently, the adipogenic induction medium was replaced with adipogenic maintenance medium (AM medium) containing α-MEM supplemented with 10 µg/ml insulin and HsMSCs were cultured for another 24 h. Next, AM medium was replaced with adipogenic induction medium, which was refreshed again at day 7 of the differentiation and exchanged for AM medium at day 10. At day 14 cells were fixed in 4% formaldehyde and stained with Nile

Red and DAPI to quantify lipid vacuoles and nuclei. The images where analyzed using Cellomics ArrayScan VTI (Thermo Scientific) with a 20x 0.45NA lens and the quantification of Nile Red and DAPI signals was done using Cellomics software.

**Table S1.** Primer sequences for quantitative real-time PCR.	
ATG7	FOR : 5’- CAGTTTGCCCCTTTTAGTAGTGC -3’<br>REV : 5’-CCTTAATGTCCTTGGGAGCTTCA-3’
ATG12	FOR : 5’- GCGAACACGAACCATCCAAG-3’<br>REV : 5’-CCATCACTGCCAAAACACTCAT-3’
ATG14	FOR : 5’- CGGGACCTGGTGGACTCCGT -3’<br>REV : 5’-TCGATAAACCTCTCCCGGTCGC -3’
BNIP3	FOR : 5’- GCCATCGGATTGGGGATCTA -3’<br>REV : 5’- CCACCCCAGGATCTAACAGC -3’
COL1A1	FOR: 5’- AGGGCCAAGACGAAGACATC-3’<br>REV: 5’-AGATCACGTCATCGCACAACA-3’
CAT	FOR: 5’-TCTCACCAAGGTTTGGCCTC-3’<br>REV: 5’-CGGTGAGTGTCAGGATAGGC-3’
GABARAPL1	FOR: 5’- ATGAAGTTCCAGTACAAGGAGGA-3’<br>REV: 5’- GCTTTTGGAGCCTTCTCTACAAT-3’
GAPDH	FOR: 5’-ATGGGGAAGGTGAAGGTCG-3’<br>REV: 5’- GGGGTCATTGATGGCAACAATA-3’
MAP1LC3B	FOR: 5’- AAGGCGCTTACAGCTCAATG -3’<br>REV: 5’- CTGGGAGGCATAGACCATGT -3’
SOD2	FOR: 5’- GGAACAACAGGCCTTATTCCAC-3’<br>REV: 5’- AGAGCTTAACATACTCAGCATAACG-3’
PARK2	FOR: 5’- CCCTGGGACTAGTGCAGAATTT -3’<br>REV: 5’- CTAAGCAAATCACGTGGCGG -3’
PINK1	FOR: 5’- GACCTTTGCCCCTAACACGA-3’<br>REV: 5’- ACGTGCTGACCCATGTTGAT-3’
ULK1	FOR: 5’- CAAGATCGCTGACTTCGGCT -3’<br>REV: 5’- CACTGGTAGACGATGGTGCC -3’
ULK2	FOR: 5’- GTTCCAAACACCTCGGTCCT -3’<br>REV: 5’- CCAACTTGAGGAGATGGCGT -3’
PIK3R4	FOR: 5’- ACCAGATGACAAACGGGCCAGA -3’<br>REV: 5’- GCCAGCAGAGGACTCGGAACG -3’
FOXO3	FOR: 5’- CTCTGCCGGCTGGAAGAACTCC -3’<br>REV: 5’- GGGCTTTTCCGCTCTTCCCCC -3’
