# Supplementary figures and images for: Activation of autophagy by FOXO3 regulates redox homeostasis during osteogenic differentiation
Source: Autophagy. 2016 Aug 17;12(10):1804–16. doi: 10.1080/15548627.2016.1203484 (PMC5079670; doi:10.1080/15548627.2016.1203484)

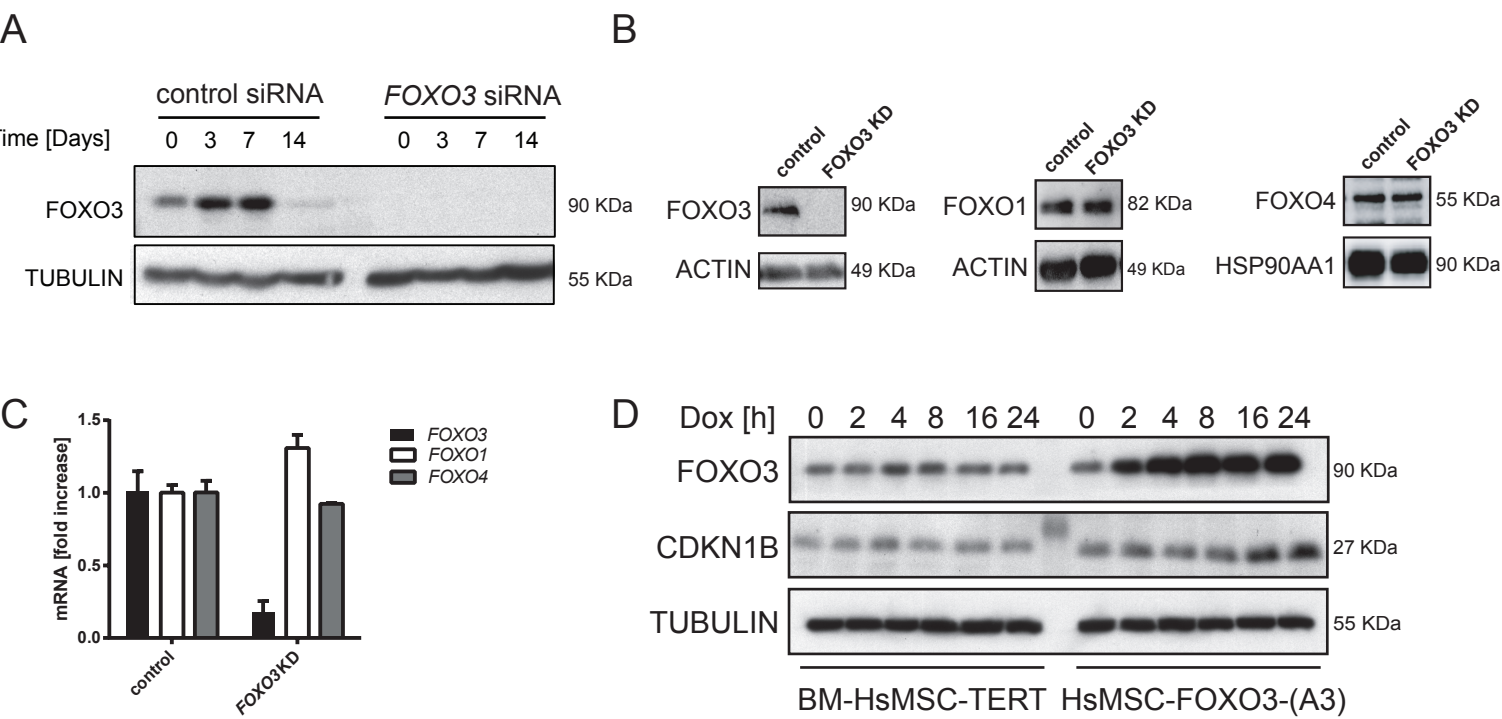

Supplement: Supplementary files [file kaup-12-10-1203484-s001.zip › 2015AUTO0617R3-s03.pdf]

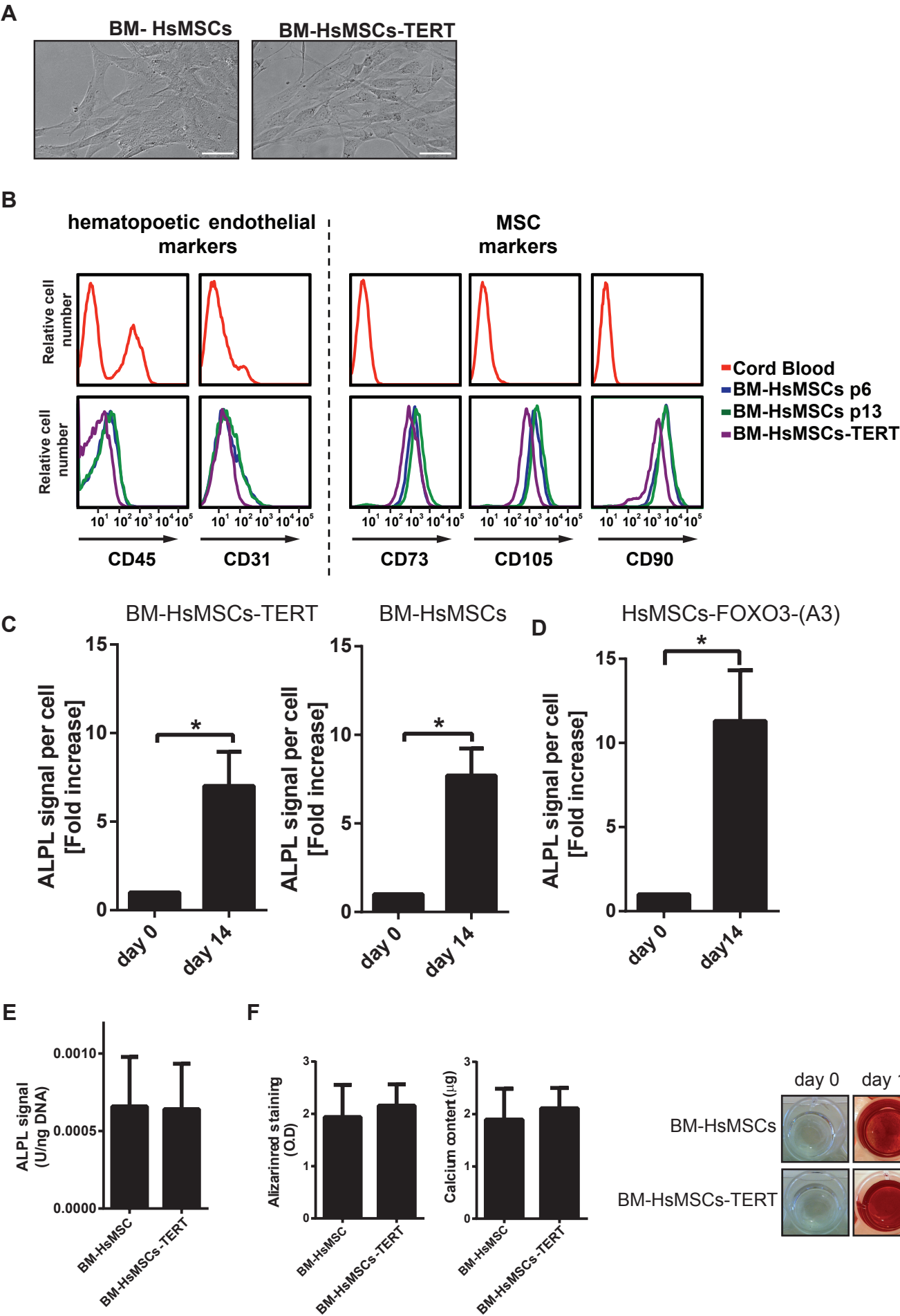

Supplement: Supplementary files [file kaup-12-10-1203484-s001.zip › 2015AUTO0617R3-s04.pdf]

A

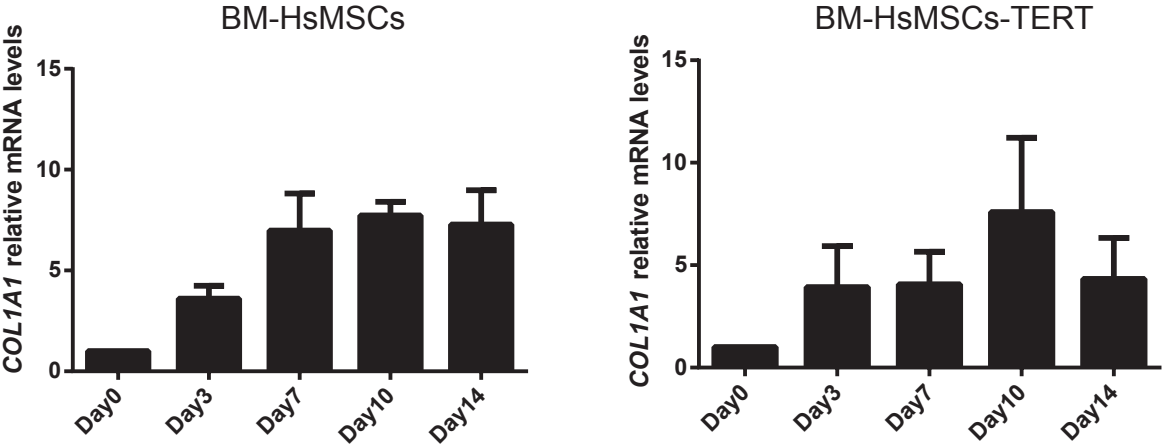

B

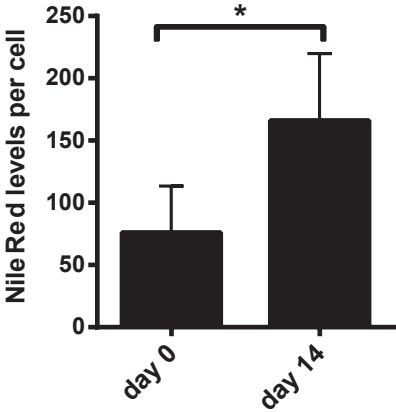

Supplement: Supplementary files [file kaup-12-10-1203484-s001.zip › 2015AUTO0617R3-s05.pdf]

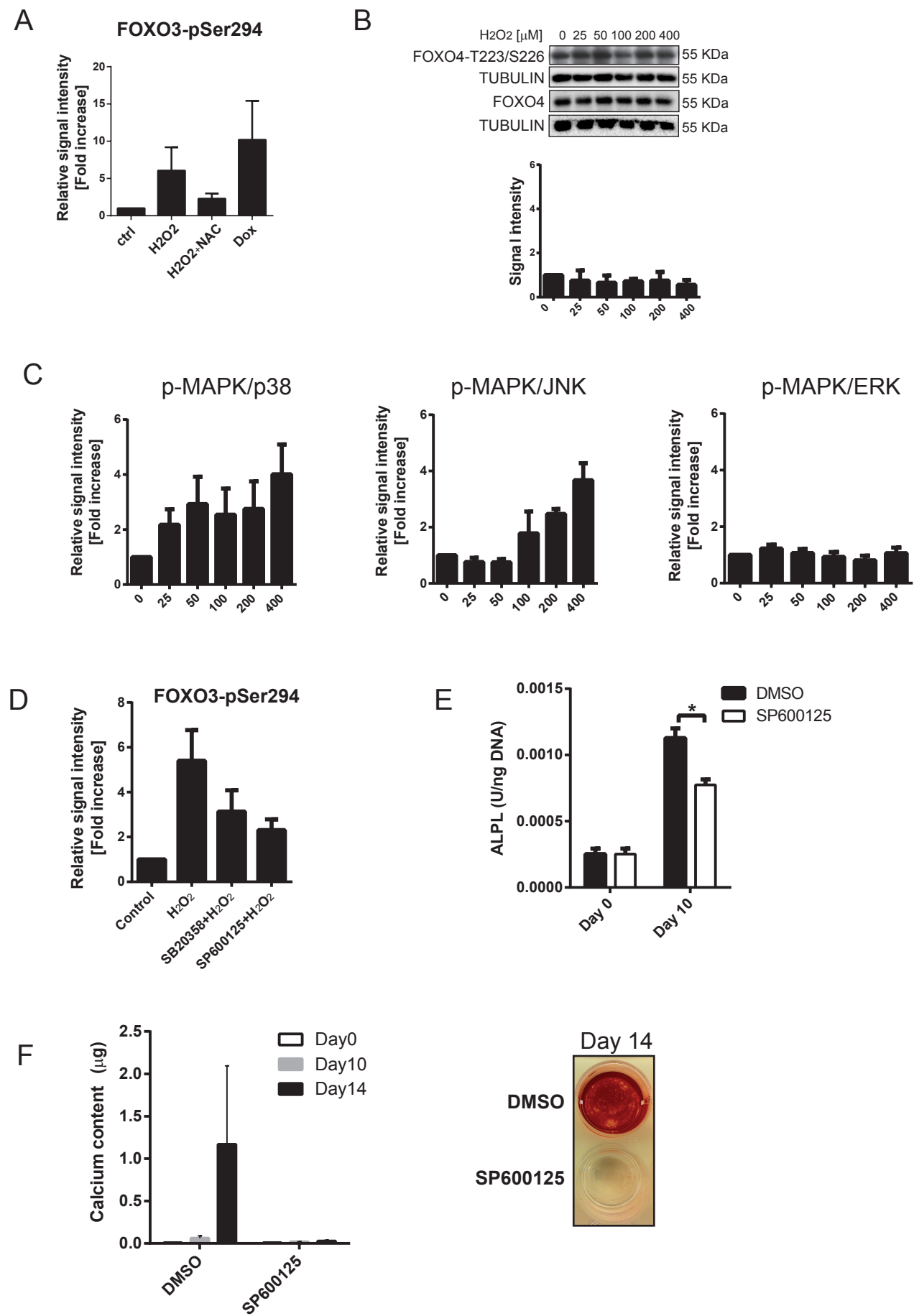

Supplement: Supplementary files [file kaup-12-10-1203484-s001.zip › 2015AUTO0617R3-s06.pdf]

## Supplementary Figure 5

Gómez Puerto et al.

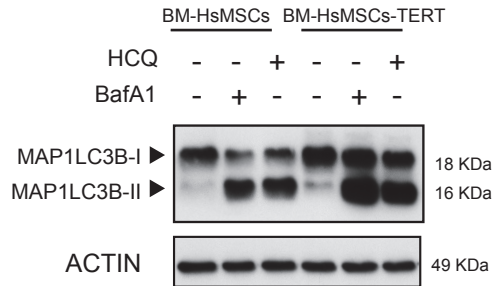

Supplement: Supplementary files [file kaup-12-10-1203484-s001.zip › 2015AUTO0617R3-s07.pdf]

A

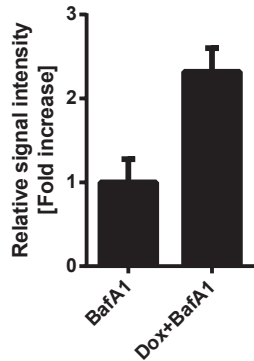

B

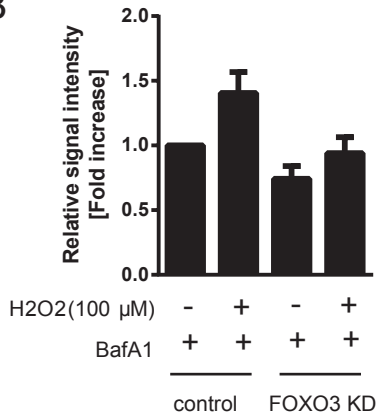

Supplement: Supplementary files [file kaup-12-10-1203484-s001.zip › 2015AUTO0617R3-s08.pdf]

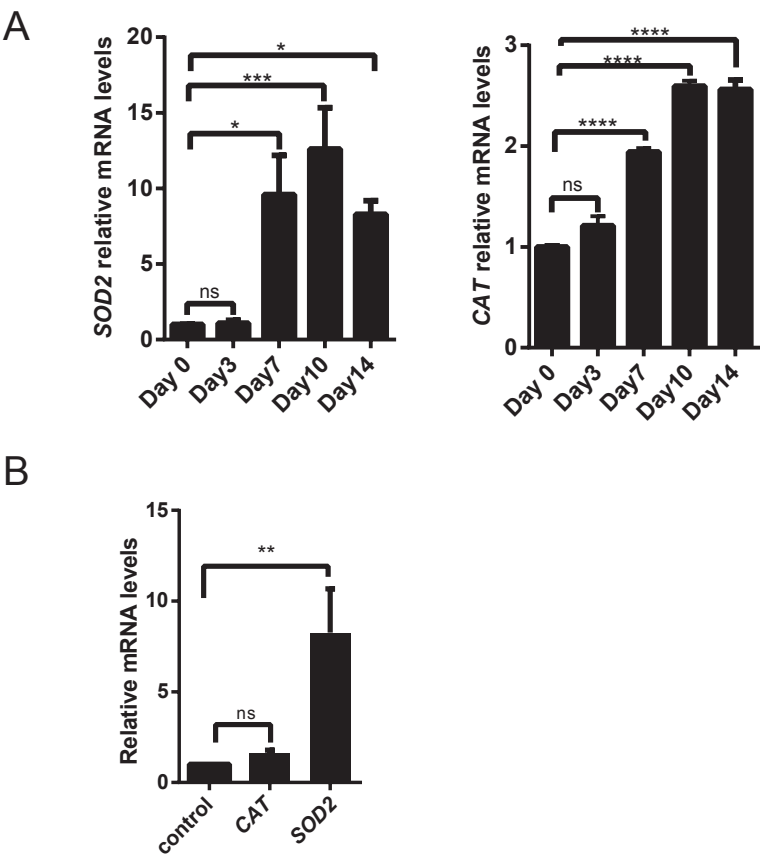

Supplement: Supplementary files [file kaup-12-10-1203484-s001.zip › 2015AUTO0617R3-s09.pdf]
